# Sensory transduction at the frog semicircular canal: how hair cell membrane potential controls junctional transmission

**DOI:** 10.3389/fncel.2015.00235

**Published:** 2015-06-23

**Authors:** Marta Martini, Rita Canella, Gemma Rubbini, Riccardo Fesce, Maria Lisa Rossi

**Affiliations:** ^1^Dipartimento di Scienze della Vita e Biotecnologie, Ferrara UniversityFerrara, Italy; ^2^Centre of Neuroscience and DISTA, Insubria UniversityVarese, Italy

**Keywords:** semicircular canal, hair cell currents, TEA effect, mEPSP release, membrane potential, receptor conductance, sensory processing

## Abstract

At the frog semicircular canals, the afferent fibers display high spontaneous activity (mEPSPs), due to transmitter release from hair cells. mEPSP and spike frequencies are modulated by stimulation that activates the hair cell receptor conductance. The relation between receptor current and transmitter release cannot be studied at the intact semicircular canal. To circumvent the problem, we combined patch-clamp recordings at the isolated hair cell and electrophysiological recordings at the cytoneural junction in the intact preparation. At isolated hair cells, the K channel blocker tetraethylammonium (TEA) is shown to block a fraction of total voltage-dependent K-conductance (IKD) that depends on TEA concentration but not on membrane potential (*V*_m_). Considering the bioelectric properties of the hair cell, as previously characterized by this lab, a fixed fractional block of IKD is shown to induce a relatively fixed shift in *V*_m_, provided it lies in the range −30 to −10 mV. The same concentrations of TEA were applied to the intact labyrinth while recording from single afferent fibers of the posterior canal, at rest and during mechanical stimulation. At the peak of stimulation, TEA produced increases in mEPSP rate that were linearly related to the shifts produced by the same TEA concentrations (0.1–3 mM) in hair cell *V*_m_ (0.7–5 mV), with a slope of 29.8 Hz/mV. The membrane potential of the hair cell is not linearly related to receptor conductance, so that the slope of quantal release vs. receptor conductance depends on the prevailing *V*_m_ (19.8 Hz/nS at −20 mV; 11 Hz/nS at −10 mV). Changes in mEPSP peak size were negligible at rest as well as during stimulation. Since ample spatial summation of mEPSPs occurs at the afferent terminal and threshold-governed spike firing is intrinsically nonlinear, the observed increases in mEPSP frequency, though not very large, may suffice to trigger afferent spike discharge.

## Introduction

A number of different approaches have been used to study the function of vestibular hair cells in processing the mechanical stimulus and producing the activation of the first afferent neuron. No experimental approach has been able, so far, to quantitatively correlate the single steps of this complex sensory processing. The electrophysiological (Ohmori, 1985; Holton and Hudspeth, 1986; Crawford et al., 1991; Martini et al., 2000, 2009, 2013; Ricci et al., 2003) and structural properties (Corey and Hudspeth, 1979; Gillespie and Müller, 2009; Kim and Fettiplace, 2013; for a review see Fettiplace, 2009) of the transduction channels, and the resulting receptor currents and receptor potentials, have been extensively studied in several hair cell types. The complex interplay of ionic currents at the isolated hair cell has also been described in detail (Housley et al., 1989; Lang and Correia, 1989; Masetto et al., 1994; Rennie and Correia, 1994; Brichta et al., 2002; Catacuzzeno et al., 2003; Martini et al., 2009), and a number of categories of sensory units have been discriminated, based on electrophysiological properties and spike firing patterns (Wilson and Melvill Jones, 1979; Goldberg, 1991; Goldberg and Brichta, 2002; Pfanzelt et al., 2008). As regards the cellular and junctional mechanisms in the intact vestibular apparatus, direct information on hair cell properties has been obtained through intracellular recording from amphibian saccular hair cells (Bracho and Budelli, 1978) at rest, whereas quantal emission and spike discharge in single afferent units, both at rest and during mechanical stimulation, have been thoroughly characterized by recording from the frog posterior semicircular canal, close to the cytoneural junction (Housley et al., 1989; Rossi et al., 1989, 1994, 2010). Only type II hair cells are present in this preparation, and they are in the physiological milieu, with the apical membrane in contact with the endolymph and the basolateral membrane surrounded by a perilymph-like solution. However, the presence of the cupula prevents intracellular recordings from single hair cells, so that their actual resting membrane potential and the size of the receptor potential during canal stimulation are unknown. The semicircular canal cupula-endolymph system is tuned for low-frequency signals, so that the type and kinetics of voltage-dependent channels in the hair cell may differ from those previously described in saccular or cochlear hair cells.

In this study we attempt to partially circumvent the gap between single-cell and synaptic studies. The idea is to find an experimental tool to produce a change in hair cell membrane potential of known magnitude and investigate the corresponding effects on synaptic activity. We show here that the K-channel blocker, tetraethylammonium (TEA), produces a fixed, voltage independent, fractional block of potassium current, so that its action on hair cell membrane potential, at a given concentration, can be mathematically predicted based on the complete characterization of the electrical properties of the isolated hair cell and of its ionic conductances, carried out in previous works (Martini et al., 2000, 2009, 2013), and experimentally verified at the isolated hair cell. The magnitude of the receptor conductance that would produce similar shifts can be estimated. In parallel, the effects of the same concentrations of TEA on synaptic activity in the intact preparation are measured in the experiments here presented, by intracellularly recording close to the posterior canal cytoneural junction at rest and during rotation. Thus, presynaptic potential and receptor conductance changes can be quantitatively correlated with the changes in the intensity of quantal release at the cytoneural junction and spike firing at the first afferent neuron.

## Materials and Methods

Frog labyrinth preparation, stimulation and recording procedures have been described in detail previously, both for patch clamp experiments (Martini et al., 2000, 2009, 2013), and intracellular recordings (Rossi et al., 1989, 1994).

All procedures for animal handling and surgery were approved by the Animal Care and Use Committee of the University of Ferrara. The experiments were performed on wild frogs (Rana esculenta, 25–50 g body weight) purchased from authorized dealers.

The frogs were anesthetized in tricaine methane sulfonate solution (1 g/l in tap water) and subsequently decapitated.

### Patch Clamp Experiments

#### Cell Preparation

Dissection of the two labyrinths was performed in a solution of the following composition (mM): 120 NaCl, 2.5 KCl, 0.5 EGTA, 5 4–2-hydroxyethyl-1-piperazineethanesulfonic acid (HEPES), 3 glucose, 20 sucrose. The final pH was 7.2 and the osmolality 256 mOsmol/kg. The six ampullae were treated for a period of 10–20 s with subtilisin A, type VIII (50 μg/ml, Sigma); the protease was thereafter blocked by trypsin inhibitor type II-S (Sigma) added to the dissection solution (final concentration 0.7 mg/ml). The ampullae were transferred to the experimental chamber (500 μl volume), submerged in the standard extracellular solution (mM): 120 NaCl, 2.5 KCl, 2 CaCl_2_, 1 MgCl_2_, 22 glucose, 5 Tris buffer—pH 7.3, in the presence of the trypsin inhibitor (osmolality 260 mOsmol/kg). Hair cells were mechanically dissociated from the ampullae by gently scraping the epithelium with a fine forceps. The protease inhibitor was eventually washed out before starting with electrophysiology. The glass bottom of the experimental chamber was coated with chloro-tri-*n*-butyl-silane to prevent cell sticking.

Cells were viewed through a TV monitor connected to a contrast enhanced video camera (T.I.L.L. Photonics, Planegg, Germany) and continuously superfused with the extracellular solution. The camera was coupled to an inverted microscope (Olympus IMT-2, Tokyo, Japan) equipped with a 40x Hoffman modulation contrast system.

#### Whole Cell Recording

Macroscopic currents were recorded at room temperature, within 1 h after cell dissociation, by using the patch-clamp technique (EPC-7, List-Electronic, Darmstadt, Germany) in the “whole-cell” configuration. Pipettes were pulled from 50 μl glass capillaries (Drummond, Broomall, PA, USA) and fire-polished to a pipette resistance of 4–5 MΩ. The pipette was filled with the following (mM): 110 KCl, 2 MgCl_2_, 8 ATP (K salt), 0.1 GTP (Na salt), 5 EGTA-NaOH, 10 Hepes-NaOH (pH 7.2; 256 mOsmol/kg). Cadmium chloride (0.2 mM) was used as blocker of voltage-dependent calcium channels, when needed. It was applied by rapidly changing (typically <50 ms) the external solution by horizontally moving a multi-barrelled perfusion pipette positioned in front of the recorded cell by means of a computer-controlled stepping motor. The same perfusion technique was employed to apply TEA-Cl (0.05–30 mM) or a mix of Cd^2+^ and TEA solution.

Isolated hair cells exhibited high membrane resistance (>1 GΩ) at –70 mV. These values indicate that no major conductances, such as hyperpolarization-activated currents or depolarizing currents due to transduction channels possibly open at rest, were significantly active in the native hair cell. Series resistance ranged 8–22 MΩ. The cell capacitance and series resistance were electronically compensated (50–75%) before running each voltage-clamp protocol. The uncompensated series resistance component introduced an error in the applied voltage command: such error was limited to 10–12 mV when the largest currents were recorded and was not corrected for. Leak was measured near resting potential with a 10 mV × 15 ms hyperpolarizing pulse and subsequently subtracted, assuming a linear behavior, in correcting current recordings off-line. Currents were low-pass filtered at 5 kHz and acquired on-line at 10 kHz with pClamp hardware and software (pClamp 9.1 and Digidata 1322A interface; Axon Instruments, Union City, CA, USA). Data were analyzed off-line by using pClamp 9.1 software. They were not corrected for the liquid junction potential, estimated to be about +4 mV under our standard recording conditions (Martini et al., 2009).

### Intracellular Recordings

Experiments were performed on the isolated and intact frog labyrinth. The frog head was pinned down at the bottom of the dissection chamber and submerged in a dissection solution containing (mM): 120 NaCl, 2.5 KCl, 2 CaCl_2_, 1 MgCl_2_, 5 glucose, 5 Tris-HCl buffer (pH 7.3); 245 mOsmol/kg. The labyrinth was isolated. The posterior canal was exposed with its nerve in the right half of the frog head; then the labyrinth, protected by the remaining bone, was separated from the head, transferred to a small Perspex chamber mounted at the center of a small turntable and fixed to the bottom of the chamber. The turntable assembly, designed for electrophysiological examination of the labyrinthine function, included a custom-made miniaturized microelectrode amplifier (0–5 kHz bandwidth) and was driven by a servo-motor controlled by a function generator. The preparation was accurately positioned so that the posterior canal lay in the plane of table rotation. Mechanical stimulation experiments were performed by subjecting the canal to a sinusoidal angular velocity stimulus at 0.1 Hz, peak acceleration 12.5°/s^2^. Intracellular recordings were obtained at rest and during rotation using glass microelectrodes (30–40 MΩ resistance), filled with 3 M KCl, inserted into the posterior nerve within about 500 μm from the synapse. The recorded potentials were transferred through low-noise sliding contacts to the oscilloscope, and stored on tape. Recordings were performed from a single fiber before and after gently substituting the TEA-containing medium for the control solution. The afferent discharge recorded from single fibers of the posterior nerve was examined off-line both in terms of spike frequency and of frequency and waveform of miniature excitatory post synaptic potentials (mEPSPs, see below, “Quantal Analysis” Section).

### Quantal Analysis

Recordings from the primary afferent axon at the frog posterior semicircular canal display quantal synaptic events (mEPSPs) (Rossi et al., 1994), which occur at a variable rate and generate action potential firing at variable frequency. At rest, about 20–30% of the units do not display any spike and when the spikes are present, their rate very rarely exceeds 10/s. When the preparation is sinusoidally rotated, both mEPSP and spike rates are clearly modulated by rotational acceleration.

We developed in the past an original procedure to analyze quantal transmitter release at the cytoneural junction: mEPSP rates of occurrence above 100/s can be measured (Rossi et al., 1989, 1994). The procedure, which was previously used to analyze cytoneural transmission in various experimental conditions, is based on the following steps:

(i) identification of the general waveform of the mEPSP, by means of fits to the spectral composition and autocorrelation of synaptic recordings: 2nd- to 5th-order autoregressive fits of the autocorrelation are used to estimate the gamma-distribution function best-fitting the “average” event waveform: $w(t)=h\cdot \frac{{\left(\beta t\right)}^{\gamma }}{\Gamma (\gamma +\text{1})}\cdot \text{exp}(-\beta t)$ , where *h* is a size factor (mV), *β* is a rate constant (s^−1^), *γ* is a numeric coefficient that determines the shape of the waveform (from a single exponential, for *γ* = 0, towards a Gaussian bell, for *γ* > 10), and Γ is Euler’s gamma function; (ii) computation of a Wiener filter from such elementary waveform; (iii) application of the Wiener filter to turn the noisy electrophysiological junctional trace into a spiky recording; and (iv) automated counting of the events.

Often the mEPSP frequencies were very high in the experiments here reported (several hundreds/s), and simple Wiener filtering, after deleting the spikes, was insufficient to accurately measure the rate of occurrence of mEPSPs from the noisy recording. We therefore employed a recently developed refinement of the procedure (Rossi et al., 2010), which combines the above mentioned Wiener filtering routine for event detection with least-square-errors optimization of fit and identification of possibly neglected events; this procedure yields the best fitting set of parameters (time of occurrence, *t_0_*, size factor, *h*, and waveform parameters *β* and *γ* for each event); events with particularly small amplitude or aberrant waveform can be excluded as artifacts.

**Figure 1:**
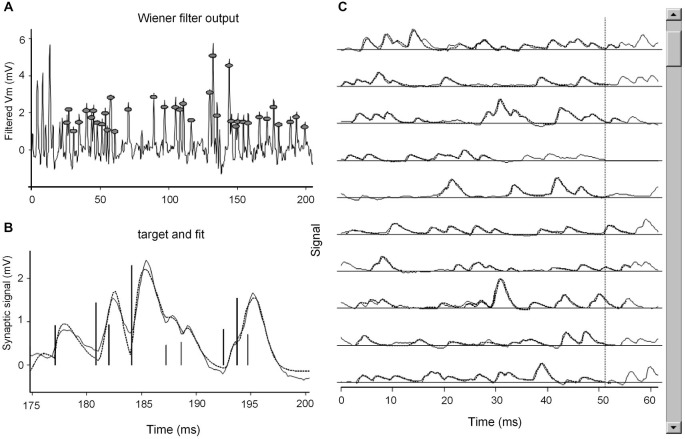
**A snapshot of the computer display during fitting. (A)** A section of the recording (200 ms) after Wiener filtering. The first 25 ms overlap the previous section and are not considered for fitting. Gray circles highlight the peaks in Wiener filter output, which are considered as indicators of the occurrence of mEPSPs. **(B)** The subsection of the record currently being fitted: the continuous line reproduces the data, the dotted line is the fit obtained by convolving the delta functions corresponding to the occurrence and size of each mEPSP (vertical bars) by analytic gamma-functions (see Section Materials and Methods), with best fitting duration (***β***) and shape (***γ***) parameters. **(C)** Continuously updated display of the overall fit: continuous and dotted lines reproduce the signal and fit; in the original display on the screen, different colors illustrate the sections just fit and currently being fitted (here, the fourth line); the cursor on the right hand side monitors the progress of fitting along the datafile.

The sequence of time intervals between successive events was translated into a continuous function that described the rate of occurrence of mEPSPs as a function of time. An example of the power of this analysis is shown in Figure 1.

This approach could be used to measure high event rates (mean mEPSP rate 400/s, instantaneous rate up to 800/s), even in the presence of quite variable size and waveforms of the individual events.

In the presence of spikes, the latter were automatically detected, counted and subtracted from the recording before proceeding with the analysis of mEPSPs. Subtraction of each spike left a 3 ms portion of the recording which could not be corrected and analyzed. This implied that some elementary events were unavoidably missed. Analysis of mEPSP rates at times immediately preceding or following the spikes, the observation that momentary rates up to 800 mEPSPs/s could be occasionally estimated in spike-free portions of the recordings, and the consideration that the coincidence of several mEPSPs must occur to generate a spike, led us to estimate that 4–6 mEPSPs were missed for each subtracted spike. Therefore, mEPSP counts were corrected by adding five mEPSPs in the 3 ms interval corresponding to each subtracted spike (Rossi et al., 2010).

In some experiments tricaine 10^−4^ M was added to the bath. The effect was complete within 2–3 min: both the resting and mechanically evoked spike discharges were abolished. On the contrary, mEPSP emission rate remained unaltered and regularly modulated by the excitatory and inhibitory phases of the sinusoidal rotation. The effect of tricaine was completely and rapidly reversible upon returning to the normal Ringer solution. This property was also used to demonstrate the reliability of the mEPSP automated counting (and spike subtraction) procedure before and after tricaine (Rossi et al., 2010).

The analysis of mEPSP size was performed on the peak amplitude of events; in particular, the time to the peak is *t*_*p*_ = *γ/β* and the peak amplitude of the event waveform is:

$P=w(t_{p})=h\cdot \frac{{\left(\beta t_{p}\right)}^{\gamma }}{\Gamma (\gamma +\text{1})}\text{exp}(-\beta t_{p})=h\cdot \frac{\gamma^{\gamma }}{\Gamma (\gamma +\text{1})}\text{exp}(-\gamma )$ (Rossi et al., 1994).

All routines for quantal analysis have been developed in Matlab software environment (Version 5.0.1 or 10a. The Math-Works, Natick, MA, USA).

### Minimal Electrical Model of the Hair Cell

The delayed outward “non-inactivating” current, IKD, in the frog hair cell was assumed to obey a Hodgkin-Huxley paradigm, controlled by a voltage-dependent activation parameter, *a*. Previous work has shown that the time constant of IKD activation is in the order of 1 ms (Martini et al., 2009), i.e., much faster than the changes in membrane potential produced by rotational stimulation at 0.1 Hz; thus, we might neglect the time-dependent transients and concentrate on the steady state value of the activation parameter of IKD, as a function of membrane potential. The parameters of a standard Boltzmann equation fitting IKD conductance over the voltage range of interest have been evaluated by fitting the average current waveform evoked in 50 experiments by a sinusoidal voltage command (−70 to −10 mV), assuming a potassium ion equilibrium potential (“zero-current potential” for potassium currents, *E_K_*) of −95 mV.

The relevant equations are: *I_KD_* = *g_KD_*·(*V_m_*–*E_K_*), *g_KD_* = *g_MAX_* · (1 + exp(−(*V*−*V_C_*)/*K_V_*)^−1^. where *g_KD_* and *g*_MAX_ indicate the total cell *KD* conductance at each value of membrane potential (*V*) and its maximal value, respectively; *V_C_* is the “center voltage”, i.e., the value at which *g_KD_* equals *g*_MAX_/2; *K_V_* is a slope parameter that determines the steepness of the dependence of *g_KD_* on membrane potential.

The non-voltage-dependent conductances are lumped in our computations into a nominal “leak” current, defined by the parameters *E*_leak_ = −68.92 mV, *g*_leak_ = 0.76 nS (Martini et al., 2009).

The delayed potassium current was modeled as a single lumped current, IKD, resulting from the summed voltage-dependent potassium current (IKV), calcium current (ICa) and voltage-calcium-dependent potassium current (IKCa); justification for doing so is that the components comprising the compound current behave functionally as a single complex, in which the physiological role of the single components is not easily isolated (Martini et al., 2009); due to the quite slow changes in membrane potential here considered, the transient potassium current (IA) gives a negligible contribution, as it is mostly inactivated at the −50 mV holding level (*h*_A∞_ = 0.07) and its activation and inactivation kinetics proceed at comparable speeds during voltage fluctuations at 0.1 Hz. The voltage dependence of IKD and its inhibition by various TEA concentrations were examined both during a sinusoidal cycle between −70 and −10 mV and in response to 300-ms voltage steps.

### Curve Fitting

The dose-response curves for inhibition of outward currents were fitted with equations of the form:

(1) $$I=I_{0}\cdot \left(\text{1}-M\cdot {\left(\frac{\left[TEA\right]}{[TEA]+K_{i}}\right)}^{h}\right)$$

where [TEA] is the concentration tested (mM), *K_i_* is the estimated affinity of the drug (mM), *M* is the maximal amount of block and *h* is Hill’s coefficient.

In experiments where the currents were measured in the presence of various concentrations of TEA while the membrane potential was slowly driven along a sinusoidal path, the current blocked by TEA was fitted using the following equation, adapted to introduce the voltage dependence:

(2) $$I_{TEA}\left(V,[TEA]\right)=I_{TEA}\left(V,[\infty ]\right)\cdot {\left(\frac{\left[TEA\right]}{[TEA]+K_{i}}\right)}^{h}$$

where *K_i_* and *h* have the same meaning as above, and *ITEA*(*V*, [∞]) represents the maximum current that can be blocked by TEA at each membrane potential value, *V*.

### Statistical Analysis

All data are reported in the text and in the figures as means ± SE. Comparisons among different conditions were performed by Student’s *t*-test (paired *t*-test for paired observations). Values of *P* < 0.05 were considered significant. Nonparametric comparisons are not reported: in each experimental series, at least five units were studied for each TEA concentration, by comparing measurements before/after applying TEA in the same unit: the changes were always consistent (decreased current in the hair cell, increased mEPSP frequency), which yields a nonparametric *P* = 2^−5^ < 0.05 in all comparisons.

## Results

**Figure 2:**
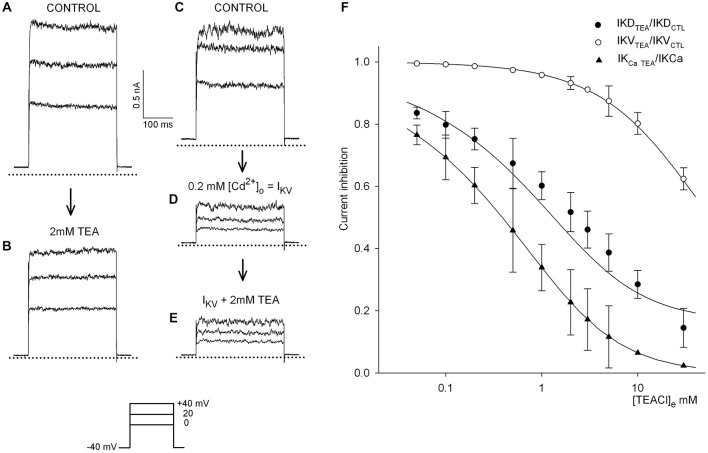
**Differential TEA blockade of the hair cell potassium currents. (A,B)** The effect of external 2 mM TEA on the delayed potassium current, IKD, evoked by voltage steps in the 0/+40 mV range, from −40 mV holding potential, in a representative frog hair cell. **(C–E)** Isolation of IKV: after blockade of the calcium inflow with 0.2 mM cadmium **(D)** 2 mM TEA does not affect the calcium-independent IKV **(E)**, evoked by the same protocol. Dotted lines indicate zero current level. **(F)** Concentration-inhibition curves: IKD peak amplitude was measured in step tests at +40 mV, in control saline and after fast TEA application; TEA inhibition was evaluated as IKD_TEA_/IKD_CTL_; data from 5–8 records in different cells were averaged for each TEA concentration (filled circles). IKV was isolated in the presence of external 0.2 mM Cd^2+^; TEA was then applied at different concentrations and its blocking effect was evaluated as IKV_TEA_/IKV_CTL_ (open circles; *n* = 4–7 for each TEA concentration). IKCa amplitude was indirectly estimated in each IKD tracing of the figure (filled circles) by considering that IKV represents the 39.9 ± 3.3% of the whole compound IKD (*n* = 31) and that IKV is unaffected by TEA at concentrations lower than 5 mM. Inhibition was thus estimated as: (IKD_TEA_−0.40 · IKD_CTL_)/(0.60 · IKD_CTL_) (triangles). The continuous lines were fitted to the data as described in the text. Note that the apparent IC50 is 19 mM for IKV and 0.28 mM for IKCa. Holding potential was −40 mV throughout. Bars indicate the SE of the mean value of the current peak amplitude.

Primary goal of the present work was to identify a pharmacological instrument to selectively block to a known (possibly voltage-independent) extent the potassium currents that physiologically balance the depolarizing transduction current. The IKCa fraction could be indirectly blocked by Cd^2+^, which impairs calcium inflow, but its effect is difficult to control in order to obtain a continuous dose-response relationship. The wide-spectrum K^+^ channel antagonist, TEA, conversely, is supposed to effectively block the IKCa, and possibly other components with lower potency: we therefore investigated the sensitivity of the different K^+^ channel types of the isolated hair cell to this agent. Figures 2A,B shows representative tracings of the effect of 2 mM TEA on the compound IKD, evoked in the 0/+40 mV voltage range; Figures 2C–E illustrates that 2 mM TEA has no effect on the calcium-independent IKV, as isolated by blocking IKCa in 0.2 mM Cd^2+^. In Figure 2F, the inhibition of each current by TEA was normalized to control conditions for each cell. Five to eight cells were tested for each TEA concentration, in the range 0.05–30 mM; the effect of TEA was assessed at +40 mV, close to the calcium ion equilibrium potential (as determined from previous analyses of calcium current I/V curves: Martini et al., 2000) to minimize any contamination of IKD amplitude by possible calcium currents (filled circles). The extent of the concentration dependent inhibition of IKD by TEA was estimated as IKD_TEA_/IKD_CTL_ (where IKD_CTL_ indicates the values measured in the absence of TEA).

To evaluate the effect of TEA on the single components, IKV was isolated by adding external 0.2 mM Cd^2+^, and the blocking effect of the same concentrations of TEA was similarly computed (IKV_TEA_/IKV_CTL_, open circles in Figure 2F; *n* = 4–7 for each TEA concentration): the effect of TEA on IKV appears to be irrelevant for concentrations <5 mM.

In these experiments, and in agreement with previous observations on higher numbers of hair cells, IKV accounted for 40% of the total compound IKD (39.9 ± 3.2% in 31 cells).

Given the insensitivity of IKV to TEA concentrations <5 mM, inhibition of IKCa by TEA was evaluated as: (IKD_TEA_ – 0.40 · IKD_CTL_)/(0.60 · IKD_CTL_) (triangles; same cell sample as for filled circles). The dose-response curves best fitting the data had the following parameters (see Eqn.1 in “Methods” Section): estimated affinity *K_i_* = 3.1 mM, maximum block *M* = 83% and Hill’s coefficient *h* = 0.43 for the IKD data (apparent IC50 = 0.71 mM); *K_i_* = 47 mM, *M* = 79% and *h* = 0.77 for IKV (apparent IC50 = 19 mM) and *K_i_* = 1.8 mM, *M* = 100% and *h* = 0.4 for IKCa (apparent IC50 = 0.28 mM). In this analysis, IKCa accounted for 63.1% and IKV for 36.9% of the whole experimental IKD, respectively. The pooled data are consistent with a half-blocking concentration for IKCa nearly 50-fold lower than for IKV. High affinity for TEA actually is characteristic of large-conductance Ca^2+^-activated K^+^ channels (Yellen, 1984), while a low affinity for TEA may indicate a purely voltage-gated K^+^ current, IKV.

In previous experiments we showed that IKD and IKCa currents exhibit similar activation time constant at any given potential (Martini et al., 2009, 2011), suggesting that IKCa and IKV channels open with similar kinetics during the depolarizing steps. Consistently, the time constant of the mono-exponential rising phase of IKD and IKV, calculated in the present experiments (five cells at +40 mV), yielded similar values: 1.08 ± 0.09 and 0.99 ± 0.03 ms, respectively. These figures are virtually identical to the values obtained for IKD in the presence of 2 mM TEA (1.04 ± 0.04 ms at +40 mV; *n* = 5). Therefore, the kinetics of both components of IKD were unaffected by the drug, in spite of its effect on IKD size.

The data above on TEA effect have been obtained by stepping membrane potential from the −40 mV holding potential to +40 mV; it was important to verify whether the drug was equally effective at any voltage. As previously reported (Martini et al., 2009), IKD machinery displays a voltage-dependent mechanism of partial steady-state inactivation. Inactivation is fully developed at −40 mV; it is completely removed (slowly) at −100 mV; this results in an increase of the evoked currents by a factor ranging 1.4–2, from the normal resting potential (−70 mV) to −100 mV. The coefficient of inactivation removal (ratio of IKD amplitude evoked from −100 or −40 mV holding potentials) was 1.63 ± 0.09 in control and 1.85 ± 0.18 after exposure to 1 mM TEA (*n* = 5; difference not statistically significant in paired Student’s *t*-test). This indicates that inhibition of IKD by TEA is independent of the inactivation machinery of this conductance. We next verified whether TEA block is also independent of the momentary membrane potential.

### TEA Effect During Sinusoidal Voltage Shifts

**Figure 3:**
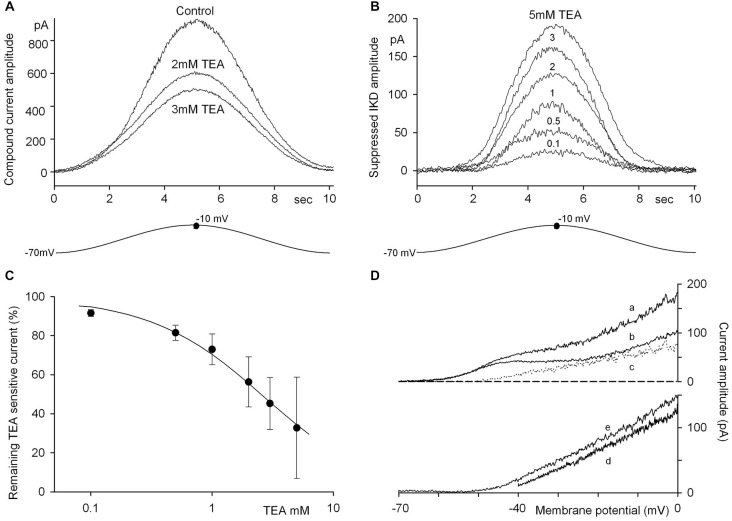
**Dissection of the current components suppressed by TEA. (A)** Current tracings recorded during a single cycle of 0.1 Hz sinusoidal stimulation (−70/−10 mV range; the precise −10 mV level is indicated by the dot) in an isolated hair cell before and after fast perfusion with 2 or 3 mM TEA. **(B)** The difference current before and after TEA application isolates the time course of the IKD current fraction suppressed by the blocker. Mean tracings of the IKD canceled by various TEA concentrations are illustrated (*n* = 7–10 for each mean recording). **(C)** Remaining current amplitude, normalized, after TEA application at the concentrations shown in **(B)**. Data points are fitted by the equation indicated in the text to describe the TEA inhibition, with *K_i_* = 3.25 mM, *h* = 0.84 and an apparent IC50 = 2.5 mM. **(D)** Tracings recorded in a single cell during ramp voltage commands of 1 s duration in the −70/0 mV range before (a) and after (b) 2 mM TEA application. The difference current (c) shows that this procedure cancels the evident contaminating currents (mainly IA, and ICa) concomitantly activated by the depolarization. Mean difference currents (*n* = 4; 5 mM TEA) evoked by voltage ramps to 0 mV starting from a holding potential of either −40 mV (d) or −70 mV (e) indicate that the suppressed K^+^ conductance is constant over the range tested. Dashed line indicates the 0 current level.

The effects of TEA were examined under conditions which more closely mimic, in the isolated hair cell, its physiological dynamic behavior during activity in the intact labyrinth, and in particular during mechanical stimulation. The membrane potential of the hair cell presumably fluctuates from an intermediate, “unstimulated” and partly depolarized level, close to −40 mV (with the cilia at an intermediate position and a fraction of the receptor transduction channels open), down to some −70 mV (all channels closed by the inhibitory deflection of the cupula) and up toward 0 mV, the upper limit set by the natural equilibrium potential of the transduction current (when additional transduction channels are open during the excitatory movements of the cupula). The receptor potential time course was therefore mimicked by driving the hair cell membrane potential along a sinusoidal waveform between −70 and −10 mV at 0.1 Hz. Figure 3A illustrates representative current tracings evoked in the same cell in control saline and in the presence of either 2 or 3 mM external TEA. The “difference current” between the tracings in the presence or absence of TEA depicts the time course of the outward current fraction actually blocked by each TEA concentration at any voltage level (which we conventionally named *I*_TEA_). The mean *I*_TEA_ waveforms (currently suppressed by 0.1–5 mM TEA) are illustrated in Figure 3B, while the mean inhibitory effects of the same TEA concentrations are reported in Figure 3C. The latter data points are fitted using Eqn. 2 (“Methods” Section), with *K_i_* = 3.25 mM, *h* = 0.84, and apparent IC50 = 2.5 mM, vs. 1.8 mM, the value reported above for TEA-mediated IKCa inhibition in voltage step experiments.

**Figure 4:**
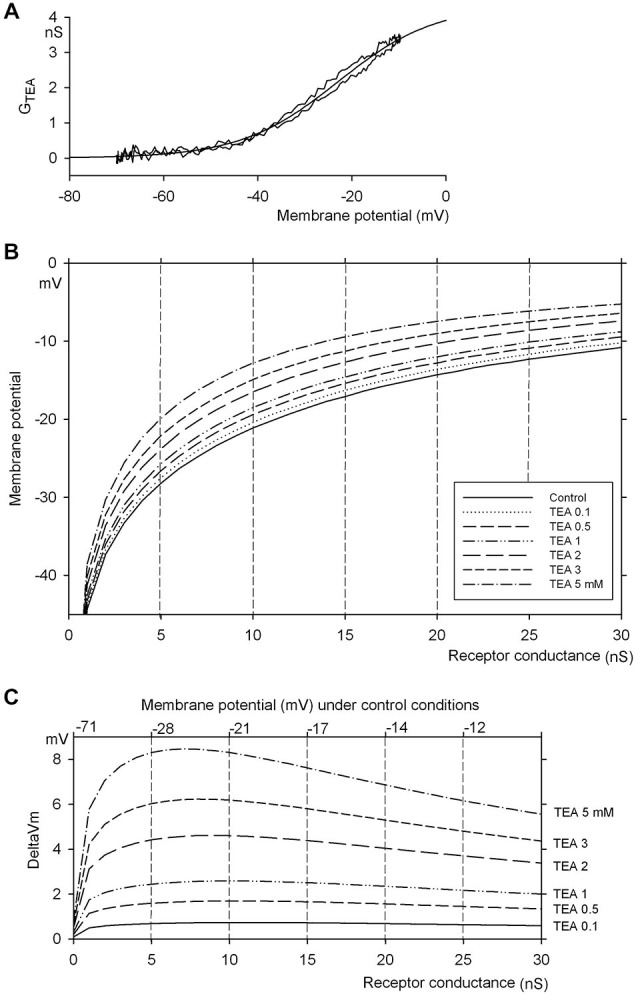
**Computation of the effect of TEA concentration on hair cell membrane potential. (A)** Mean TEA sensitive conductance vs. membrane potential curve. The continuous line was obtained by averaging the traces in Figure 3B, after scaling them up to the no-TEA condition, based on the inhibition curve in Figure 3C. The dotted line is the Boltzmann equation best fitting the data (parameters reported in the text). **(B)** Hair cell membrane null potential, computed as a function of receptor conductance, based on the estimated TEA-sensitive voltage-dependent conductance (Panel **(A)**) and a lumped voltage-independent conductance (0.76 nS, zero-current potential –70 mV, see text), in control and in the presence of increasing concentrations of TEA. Notice that the curves become almost perfectly parallel for membrane potentials above −30 mV. **(C)** shift of membrane potential produced by various concentrations of TEA (data from Panel **(B)**, after subtracting the control curve). The numbers on top indicate the zero-current potential values corresponding to the transduction conductance in abscissa, in control conditions (no TEA). Notice that the shifts are almost voltage-independent in the range −30 to −15 mV.

A full dynamic conductance-voltage relation was derived from these data by fitting the average current response to the sinusoidal voltage command. The data are illustrated in Figure 4A.

The resulting equation, describing TEA-sensitive potassium conductances, was:

(3) $$g_{TEA}(V)=\frac{G_{TEA}}{\text{1}+\text{exp}\left(-(V+\text{23}.\text{0})/\text{10}.\text{3}\right)}$$

where *g_TEA_* = 4.3 nS in control conditions, and the full dynamic behavior in the presence of TEA is well described by the equation:

(4) $$g_{TEA}\left(V,[TEA]\right)=\frac{G_{TEA}}{\text{1}+\text{exp}\left(-(V+\text{23}.\text{0})/\text{10}.\text{3}\right)}\times {\left(\frac{\left[TEA\right]}{[TEA]+K_{i}}\right)}^{h}$$

The waveforms in Figure 3A are apparently asymmetrical, when the current amplitudes during the excitatory or inhibitory acceleration phases (excitatory and inhibitory hemicycle) are compared; evaluation of the charge movements actually indicates that the total charge during the inhibitory phase is smaller than during the excitatory phase by 4–19%, in the various runs. The difference current procedure, in principle, cancels any contaminating current component insensitive to TEA, as ICa and IA actually are. The effective cancellation of TEA-insensitive components was verified more directly in experiments in which currents were observed during −70/0 mV voltage ramps. Figure 3Dab shows current tracings evoked in the same cell from a −70 mV holding level, before and after 2 mM TEA application: the profiles actually confirmed the presence of different current components (here the contribution of IA is evident, due to inactivation removal during the sojourn at −70 mV holding potential). However, the difference current, i.e., TEA-sensitive current, exhibited a clear-cut linear I-V relation above −40 mV (Figure 3Dc). The mean difference currents shown in Figure 3Dde (5 mM TEA) confirm that the contaminating components were mutually canceled by the subtraction procedure, when the ramp starting level was either −40 (d) or −70 mV (e). Moreover, their constant slopes indicate that the fraction of K^+^ conductance blocked by TEA was voltage-independent, at least over the −40/0 mV range.

The asymmetry during the rising and falling phases persisted in the difference currents and might thus be related to the activation/deactivation kinetics of the underlying channels. In Figure 3A most of the asymmetry seems to occur in the portion of the curves corresponding to *V*_m_ < −40 mV, where *I*_TEA_ is negligible. However, a slight asymmetry persisted when difference currents (*I*_TEA_) obtained at various TEA concentrations were normalized and plotted vs. *V*_m_, to produce the plot of *g*_TEA_ vs. *V*_m_ illustrated in Figure 4A. The resulting curves superimposed very well, indicating that TEA did not alter their shapes and thus confirming the voltage-independence of current block by TEA, even in the presence of asymmetry; the latter merely produced a slight hysteresis (difference between rising and falling phases) that can be perceived in the conductance vs. *V*_m_ plot (Figure 4A).

Thus, TEA constitutes a flexible, selective tool to produce the desired fractional block of IKCa under most experimental conditions.

### Electrical Effects of TEA Application

Due to the interplay of receptor and voltage-dependent potassium currents, the observed fixed fractional block of voltage-dependent potassium current induces a relatively fixed shift of membrane potential, when the cell is depolarized in the range −30 to −10 mV (i.e., in presence of strong activation of the receptor current). At rest, the cell displays a baseline conductance of about 0.76 nS, with a zero-current potential of –70 mV: this compound voltage-independent conductance can be represented by

$$C=\sum_{i=\text{1}}^{n}G_{i}$$

and gives rise to a compound, “leak” current, given by

$$I_{LK}=\sum_{i=\text{1}}^{n}\left(V-E_{i}\right)\cdot G_{i}=(V+\text{70})\cdot C.$$

In the presence of a receptor conductance (*g*_REC_ = G_0_, reversal potential ≈0 mV), the membrane potential (zero-current) can be computed as:

$$V_{m}=\frac{E_{K}g_{K}+\sum_{i}^{n}E_{i}G_{i}}{g_{K}(V)+\sum_{i}^{n}G_{i}+g_{REC}}=\frac{E_{K}g_{K}(V)-\text{70}\cdot C}{g_{K}(V)+C+g_{REC}},$$

where *g_K_(V)* represents the voltage-dependent conductances (mostly potassium currents, as above mentioned). Increasing receptor currents produce increasing depolarizations. However, given the voltage dependence of *g_K_(V)*, this will both enhance *g_K_(V)* activation and increase the driving force for K^+^ ions, which will counteract the action of the receptor current. Thus, the amount of receptor current required to depolarize the cell by a fixed step increases with membrane depolarization; on the other hand, a partial block of *g_K_(V)* would cancel an amount of potassium current that similarly increases with membrane depolarization. This suggests that, independently of membrane potential—and the momentary level of activation of *g_REC_*—a fixed fractional block of *g_KD_* might produce a similarly sized depolarization, independent of the degree of activation of receptor current and the resulting membrane potential.

A basic numerical simulation, obtained by considering the description of *g_KD_* and “leak” current derived from these experiments (see above) actually confirms this prediction.

Figure 4B illustrates the membrane potential produced by a receptor conductance from 0 to 30 nS (continuous line) and the effects of blocking 8, 18, 27, 44, 55, and 67% of *g_K_(V)*, i.e., the block experimentally measured after applying 0.1, 0.5, 1, 2, 3, 5 mM TEA, respectively. The shifts in *V*_m_ produced by such block are illustrated in Figure 4C (the numbers at the top remap the receptor conductance in abscissa to the corresponding membrane potential in the absence of TEA): it can be observed that, for g_REC_ ≥ 4 nS (i.e., a membrane potential *V*_m_ ≥ −30 mV, as expected at the peak of rotational stimulation) an almost constant shift in membrane potential is produced by each fractional block (0.69–0.73 mV for 8% block, 1.6–1.7 mV for 18%, up to 7.2–8.5 mV for 67% block), independent of the intensity of receptor current, over the range of membrane potential −30 to −15 mV (which constitutes a reasonable range for hair cell membrane potential at the peak of rotational stimulation).

This observation suggests that, even though the hair cell membrane potential (or the receptor current) cannot be measured in the intact preparation, application of a drug that can block a fixed fraction of *g_KD_* will produce a predictable shift in membrane potential, and the magnitude of such shift can be estimated based on the measurement of fractional inhibition of *g_KD_* in the isolated hair cell.

### mEPSP Discharge Properties in Control Units

**Figure 5:**
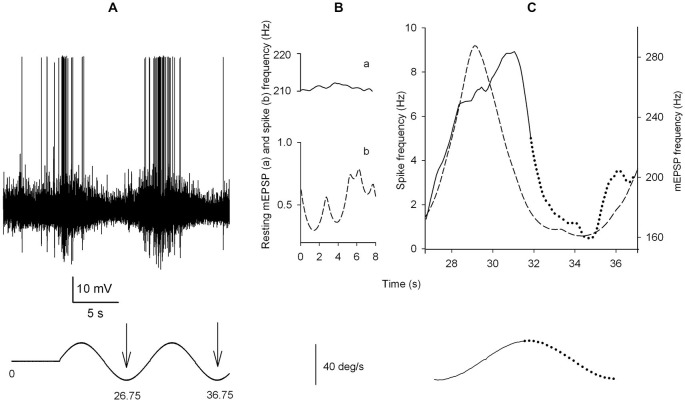
**Analysis of quantal emission and spike discharge at rest and during rotatory stimulation. (A)** Intracellular recordings from an axon during a rest period followed by rotatory stimulation at 0.1 Hz, 12.5°/s^2^. Action potentials are truncated. The lower trace illustrates turn-table velocity (same time scale). The first complete cycle used in the analyses is marked by arrows in the velocity diagram of the turn-table rotation. Notice that synaptic activity anticipates turn-table velocity, but lags behind turn-table acceleration. **(B)** mEPSP (top) and spike (bottom) rates during the initial resting recording. **(C)** mEPSP (continuous-dotted line) and spike (dashed line) rates during a full cycle of rotation, as indicated by the bottom trace and the arrows in the bottom trace of Panel **(A)**. The continuous line section, for mEPSP rate as well as turntable velocity, indicates the excitatory hemicycle, defined as the portion during which turntable acceleration is ≥0; conversely, the dotted section indicates the inhibitory hemicycle. Notice that mEPSP rate anticipates turntable velocity by about 80° (i.e., it is almost in phase with turntable acceleration); spike rate, conversely appears to anticipate some more degrees, moving in phase with turntable acceleration.

In experiments on isolated hair cells, the transmitter released by depolarization is wasted in the external saline. In the intact preparation, where the postsynaptic cell (that is the natural transmitter detector) is present, the native receptors disclose the characteristics of presynaptic quantal emission and of the postsynaptic response. A large amount of information can be extracted from a complex signal such as the afferent sensory discharge. Some parameters are presented in Table 1, as evaluated at rest or during 0.1 Hz rotatory stimulation at 12.5°/s^2^. An example of the tracings and analyses from which these data are derived is shown in Figure 5.

**Table 1:** **mEPSP properties in control preparations at rest or during sinusoidal stimulation (0.1 Hz, 12.5°/s^2^)**.

Spontaneous activity (SA; mEPSPs/s)	209 ± 21
Number of mEPSPs in the excitatory hemicycle	1409 ± 121
Number of mEPSPs in the excitatory hemicycle in excess of SA	361 ± 48
Number of mEPSPs in the inhibitory hemicycle	1048 ± 99
Peak mEPSP frequency (mEPSPs/s; excitatory hemicycle)	315 ± 23
Lowest mEPSP frequency (mEPSPs/s; inhibitory hemicycle)	136 ± 15
mEPSP Peak Size during spontaneous activity (mV; *n* = 7,856)	0.65 ± 0.005
mEPSP Peak Size in the excitatory hemicycle (mV; *n* = 17,509)	0.66 ± 0.003
mEPSP Peak Size in the inhibitory hemicycle (mV; *n* = 11,999)	0.60 ± 0.003

*n = 14, for mEPSP amplitude evaluation; n = 28 for mEPSP numbers*.

In these experiments a single stimulation pattern was tested; the following points of physiological interest can be stressed: (1) the cytoneural junction sustains a high mEPSP discharge level at rest; (2) the increment in quantal discharge rate during the excitatory hemicycle, after correction for the underlying Spontaneous activity (SA), was relatively scarce (a mean of 361 ± 48 quanta in a 5 s period—in the present example of 0.1 Hz rotatory stimulation at 12.5°/s^2^). However, if mEPSP discharge at rest brings the postjunctional membrane potential close to the spike-firing threshold, then even such a modest extra quantal release might be instrumental in determining the final sensory response that reaches the CNS (i.e., spike firing); (3) during the inhibitory hemicycle the frequency reaches a minimum that is clearly lower than the mean SA (136 ± 15 vs. 209 ± 21 quanta/s; statistically different, *P* < 0.001, paired *t*-test; *n* = 28); however the peak of inhibition during the sinusoidal stimulation here applied is insufficient to completely turn off quantal release; (4) in the average, the evoked quantal release during the whole stimulation cycle is higher than at rest; actually, during the whole inhibitory hemicycle the average rate is not lower than during the initial SA, indicating that during mechanical stimulation release is modulated about a higher level than at rest (see “Discussion” Section).

### Effect of TEA on mEPSP Discharge

We have shown that TEA treatment results in a robust decrease of the potassium current amplitude, which produces a decrease in the repolarizing capability of the hair cell, increased calcium influx and activation of the cytoneural junction. We therefore examined quantitatively the correlation between TEA concentration and mEPSP emission. The relevant parameters are summarized in Table 2, as an illustration of the effects of 1 mM TEA on the native quantal discharge.

**Table 2:** **Effect of 1 mM TEA on some mEPSP discharge properties, at rest or during sinusoidal stimulation (0.1 Hz, 12.5°/s^2^)**.

	Control	1 mM TEA	Difference
Spontaneous activity (SA) mEPSPs/s	167 ± 41	234 ± 48*	66 ± 13
Number of mEPSPs in the excitatory hemicycle	1215 ± 256	1607 ± 312*	392 ± 130
Number of mEPSPs in the inhibitory hemicycle	850 ± 193	1073 ± 216*	223 ± 69
Peak mEPSP frequency (excitatory cycle)	256 ± 42	401 ± 40*	145 ± 47
Lowest mEPSP frequency (inhibitory hemicycle)	87 ± 17	156 ± 36	69 ± 25
mEPSP Peak Size during SA (mV; *n* = 6,135 vs.1,218)	0.64 ± 0.005	0.63 ± 0.009	
mEPSP Peak Size in the excitatory hemicycle (mV; *n* = 1,297)		0.75 ± 0.01	
mEPSP Peak Size in the inhibitory hemicycle (mV; *n* = 1,092)		0.62 ± 0.009	

*Statistically different in paired t-test (P < 0.05); n = 5.

**Figure 6:**
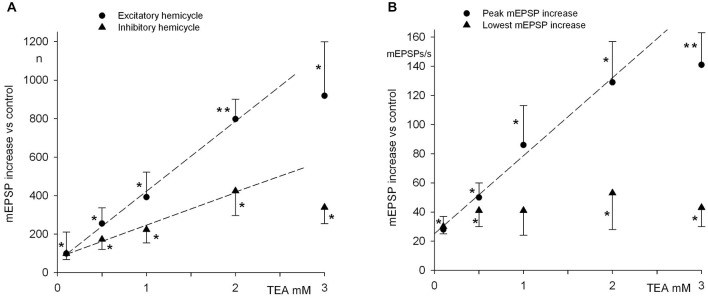
**mEPSP emission as a function of the applied TEA concentration. (A)** Plot of the increase of mEPSP numbers vs. control as a function of TEA concentration, during the whole excitatory (closed circles) or inhibitory (triangles) hemicycles. Mean data from 5–6 cells for each TEA concentration tested. TEA effect was statistically significant in paired comparisons at all TEA concentrations (*P < 0.05; **P < 0.01; paired *t*-test). The straight lines indicate the best linear fit to data for 0.1–2 mM TEA (*r* = 0.99, *P* < 0.01). **(B)** Variations of the peak (closed circles) and minimum (triangles) mEPSP frequency vs. control values during 0.1 Hz sinusoidal cycles, plotted against TEA concentration (same units as in Panel **(A)**). The straight line was fitted as in Panel **(A)**, with the same *r* and *P* values. TEA effect statistically significant where indicated (as in Panel **(A)**).

Additional results concerning TEA application in the 0.1–3 mM concentration range are summarized in Figures 6A,B; for clarity, only quantal emission rates are considered, focusing on their *variation* rather than on absolute values. In particular, two distinct measurements are presented: the variations in number of mEPSP during one—excitatory or inhibitory—hemicycle and the variations in maximum and minimum mEPSP frequencies during the stimulation cycles. The analysis was restricted to the 0.1–3 mM TEA concentration, because TEA obviously acts on the postjunctional membrane as well, and above 3 mM it produces spontaneous repetitive activity in the nerve fibers; this effect becomes so relevant that a too large fraction of the record has to be eliminated by the spike deletion procedure, and the resulting measurements of mEPSP rate become unreliable; spiking rate itself becomes little informative, under these conditions.

The increase in mEPSP numbers vs. control, during the excitatory hemicycle, linearly paralleled TEA concentration up to 2 mM TEA (Figure 6A; filled circles—correlation coefficient *r* = 0.99, *P* < 0.01). The increase was statistically significant (*P* < 0.05 in paired *t*-test) at all TEA concentrations (*n* = 5–6 for each data point). In the same cells, mEPSP numbers during the inhibitory hemicycle (Figure 6A, triangles) also exhibited an increase at all TEA concentrations (*P* < 0.05, paired *t*-test), but the concentration dependence could not be consistently evaluated (regression through data points was not statistically significant). It should be observed that our analysis above indicates that below −40 mV (i.e., during the inhibitory cycle) the IKD rapidly declines and, consequently, the shift in membrane potential produced by TEA similarly declines (see Figure 4C). The same patterns—namely, a significant concentration-dependent increase during the excitatory hemicycle and a mild decrease with little/no concentration dependence during the inhibitory hemicycle—were observed after normalizing (i.e., by considering the percentage increases in mEPSPs numbers).

As it occurred in control preparations, the minimum mEPSP frequency during the inhibitory cycle was lower than SA (Table 2), i.e., a fraction of the spontaneous discharge was suppressed during the maximal inhibitory phase of the cycle.

The variations produced by the various TEA concentrations in peak and minimum mEPSP *frequency* vs. control are shown in Figure 6B (same cells as in A). For peak values the pattern was similar to that described for the whole hemicycle *numbers*: namely an apparent linear increase with increased TEA concentration up to 2 mM (*r* = 0.99, *P* < 0.01), and a deflection from linearity at 3 mM TEA. The minimum rates were apparently independent of TEA concentration (again consistent with the idea that at the moment of maximal inhibition, the IKD are shut and little effect is to be expected from TEA).

As shown for the 1-mM example (Table 2), the mEPSP peak size was not modified by the presence of TEA, either during SA or during the hemicycles of sinusoidal stimulation, even in the presence of relatively high TEA concentration. Typically, the mean spontaneous mEPSP peak size in five units, before and after 3 mM TEA application, were 0.68 ± 0.005 (*n* = 8, 422) vs. 0.75 ± 0.005 mV (*n* = 9, 732), respectively. This difference actually is significant (*P* < 0.05); however, its magnitude is quite small, and it might be fully accounted for by small changes in postjunctional conductances, brought about by TEA, which may affect the response of the post-junctional membrane. Altogether these data indicate that possible effects of TEA on the size of the elementary quantum of transmitter, or on the sensitivity of the postsynaptic transmitter receptor, are not relevant.

### A Quantitative Correlation Between IKD Blockade and mEPSP Discharge?

Figure 3B shows the effect of TEA in suppressing a fraction of the outward potassium current, which has been quantitatively evaluated for each concentration tested. These effects were due to the block of variable amounts of the natural potassium conductance, activated by cell depolarization. The fractional block was voltage-independent in the −70 to 0 mV range (Figure 3D); during the sinusoidal voltage shifts, it was best evaluated at −10 mV, i.e., when the driving force for K currents is maximal.

As discussed above, a fixed fractional block of TEA-sensitive, IKD is expected to produce a depolarizing shift whose magnitude depends on the underlying interplay of conductances and momentary membrane potential. However, if the cell is depolarized (in the range of −30 mV or higher), as it is supposed to be at the peak of the excitatory phase of a rotation, then a fixed fractional block produces an almost constant shift of membrane potential (Table 3).

**Table 3:** **Mean gK(V) inhibition, expected shift of hair cell membrane potential and net increase in peak mEPSP frequency during stimulation, as functions of external TEA concentration**.

TEA concentration (mM)	gK(V) inhibition (%)	Membrane potential shift (mV)*	mEPSP peak frequency increase (Hz)
0.1	8	+0.66 (0.59–0.72)	28 ± 9
0.5	18	+1.50 (1.35–1.65)	50.4 ± 19
1	27	+2.25 (2.0–2.5)	86 ± 27
2	44	+3.90 (3.4–4.4)	129 ± 28
3	55	+5.1 (4.4–5.8)	141 ± 55
5	67	+6.6 (5.6–7.6)	==

*In parentheses the minimum-maximum values over the range −30 to −15 mV.

**Figure 7:**
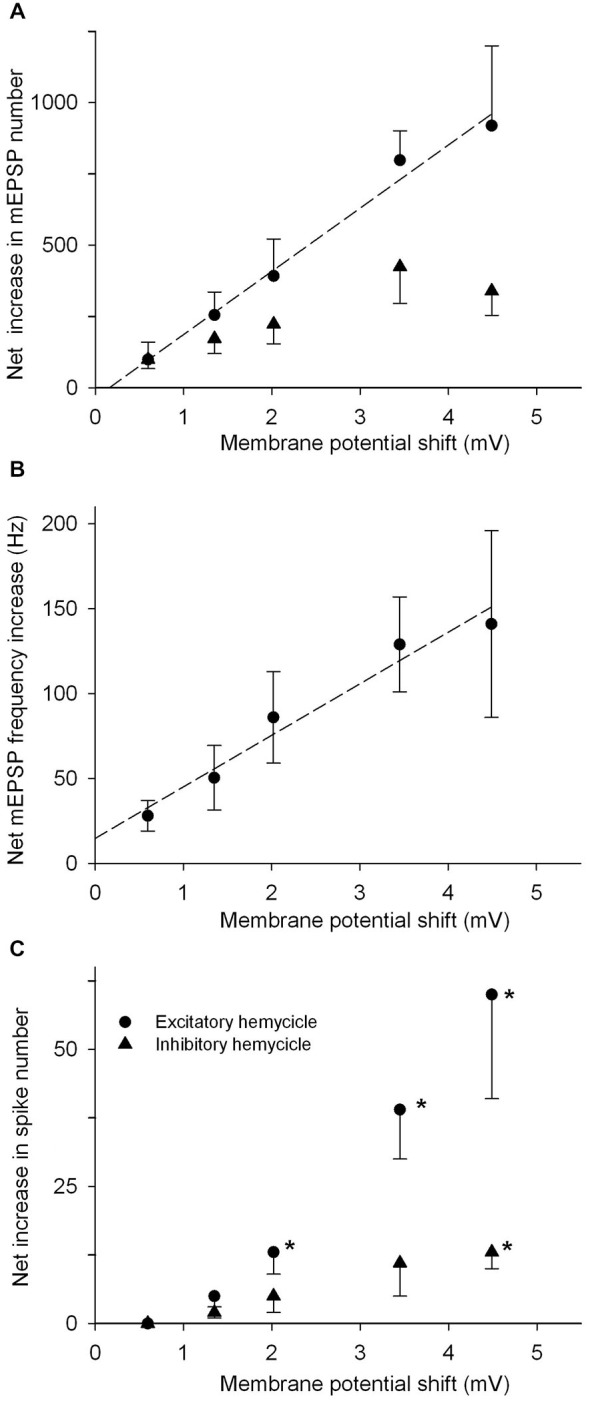
**Parameters of quantal release as a function of cell membrane depolarization. (A)** Net increase, with respect to control, of mEPSP numbers discharged during the whole excitatory (filled circles) or inhibitory (triangles) hemicycles at 0.1 Hz sinusoidal rotation, plotted against the membrane potential shifts expected from TEA application. The straight line is the best linear fit to the data (*r* = 0.99, *P* < 0.01), and indicates the sensitivity of the quantal release mechanism to membrane potential modifications (221 quanta/mV for the excitatory hemicycle). **(B)** Net mEPSP peak frequency increase vs. control as a function of membrane potential shifts. The straight line (*r* = 0.99, *P* < 0.01) suggests that a mean of 30.3/s quanta are released per mV of membrane potential variation. **(C)** Afferent spike extra discharge (vs. control) as a function of membrane potential variation, during the whole excitatory or inhibitory hemicycle. Note the non-linear relationship and the statistical significance of the effect for membrane potential shifts ≥2 mV, in the excitatory hemicycle, (*P < 0.05 in paired *t*-test); for the inhibitory hemicycle,actually, the abscissa is meaningless, as the membrane potential shifts presumably are much smaller during the inhibitory phase (see the steep drop of delta-*V*_m_ for small values of transduction conductance in Figure 4C).

The parameters of mEPSP emission are plotted in Figures 7A,B against the membrane potential shifts produced by TEA. The variations in absolute numbers of mEPSPs during the excitatory/inhibitory hemicycles (panel A) and in peak mEPSP emission frequency (panel B), are linearly related to the estimated variations in membrane potential. Statistical analysis indicates that the linear fit is quite good for excitatory responses (*r* = 0.99, *P* < 0.01 in A,B), but not for the number of mEPSP during the inhibitory hemicycle (triangles in panel A). The straight line in each panel thus represents a sort of calibration curve to transform delta-*V*_m_ into mEPSP discharge changes and vice-versa, within the range explored. Functionally, this implies that a shift of 1 mV in membrane potential generates: (1) the extra emission of 30.3 quanta/s at the peak of excitation (Figure 7B), at membrane potentials presumably more positive than –15 mV; (2) the discharge of 221 extra mEPSPs, when maintained during the whole 0.1 Hz hemicycle (Figure 7A), i.e., in a presumable membrane potential range –40 to –10 mV. The linear relation with membrane potential holds for shifts in the range 0–7 mV at least.

**Figure 8:**
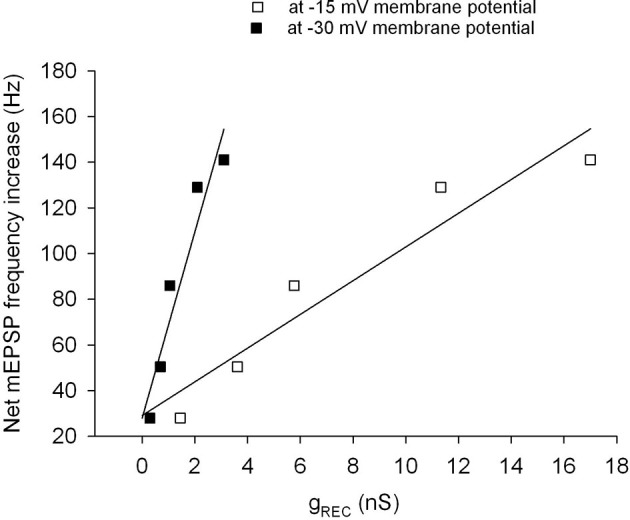
**Expected relationship between mEPSP rate and receptor conductance**. Increase in peak mEPSP rate during rotational stimulation, produced by various concentrations of TEA, plotted vs. the receptor conductance that would produce the same shift of membrane potential, assuming a maximal depolarization either to −30 mV (filled symbols) or to −15 mV (open symbols). The regression lines would indicate a sensitivity for the quantal release machinery of 7–41 quanta/s per nS of transduction conductance, depending on the maximal depolarization achieved.

Although a change in receptor conductance, *g*_REC_, produces a shift of membrane potential that depends on the latter (and on the resulting driving force across the transduction channel), the local slope d*V*/d*g*_REC_ allows us to estimate the extra emission of quanta elicited by a small change in receptor conductance: 1 nS of extra *g*_REC_ at –30 mV will increase mEPSP frequency by 40.8/s, whereas a similar change in conductance at –15 mV will produce the emission of 7.4 more quanta/s (Figure 8).

### TEA Effect on Afferent Spike Discharge

The main target of the present experiments was understanding the properties of quantal emission at the cytoneural junction and its relation with hair cell depolarization. The presence of spikes in the tracings was still compatible with a reliable estimate of the mEPSP emission (see above in “Materials and Methods” Section); nonetheless in some experiments spiking was blocked by tricaine application, and in others bias was possibly introduced by selecting the non excessively spiking units. Despite these limits, the extra spike discharge observed after TEA application deserves a functional consideration. Spontaneous spiking was in the range 1–3/s (*n* = 15) and was scarcely affected by 0.1–2 mM TEA; only at 3 mM TEA spikes numbers increased by a mean of 72% (*P* < 0.05, paired *t*-test; *n* = 6). Figure 7C indicates that the extra spike numbers during the excitatory hemicycle increased with increasing depolarization. However, the effect was not directly proportional to the membrane potential shift and only was significant above +2 mV: since spike firing is a thresholded process, this may well be due to the temporal summation of mEPSPs required to trigger a spike.

## Discussion

We studied some basic features of early sensory processing in the frog semicircular canal, a preparation in which the isolated hair cells are accessible to the patch-clamp analysis, while the afferent information can be recorded in the intact labyrinth. Owing to the presence of the cupula, single hair cells cannot be accessed electrophysiologically in the intact preparation; thus, in defining the properties of transmitter release by the hair cell its membrane potential (and its fluctuations during mechanical stimulation) must be indirectly evaluated.

### The Problem of the Hair Cell Membrane Potential

Data from the literature suggest that amphibian saccular hair cells *in situ* and *in vivo* should have a resting membrane potential around –70 mV (Bracho and Budelli, 1978) and that the receptor conductance can change the membrane potential by some 8 mV in mechanically stimulated isolated saccular hair cells (Hudspeth and Corey, 1977); in type II hair cells, a model derived from available data suggested a membrane potential fluctuation, in response to maximal cilium deflection, from −70 to −40 or −30 mV, depending on the expression of the various conductances (Soto et al., 2002); a depolarization up to −19 mV was measured in vestibular hair cells of the chick held at a resting potential of −43 mV (Ohmori, 1985).

There are no means to measure hair cell membrane potential in the intact posterior canal; however, the above cited measurements, our estimate of resting potential in isolated hair cells from the posterior canal and reasonable physiological and functional considerations suggest that the membrane potential should physiologically oscillate between about −70 mV (resting potential in isolated hair cells, no receptor current) and a maximal depolarization in the −30 to −20 mV range (at the peak of mechanical stimulation).

### Potassium Conductances and the Effect of TEA

Our previous work has indicated that most voltage-dependent current in canal hair cells is carried by potassium conductances (Martini et al., 2009). Although IA surely plays an important role in cochlear and vestibular hair cells subjected to rapid accelerations, in the face of slow mechanical rotations (0.1 Hz) and similarly slow membrane potential changes, its contribution is negligible, as it becomes inactivated as soon as it gets activated. Thus, voltage-dependent currents are dominated by the delayed potassium current, IKD, with its purely voltage-dependent component (IKV) and its voltage- and calcium-dependent component (IKCa).

We show here that external TEA selectively blocks the IKCa of the isolated hair cell in a graded manner at concentrations lower than 5 mM, and that the extent of block by TEA is fixed in the membrane potential range –70 to 0 mV. We assumed that TEA application in the intact preparation might have the same effects as on the isolated hair cell, considering that the basolateral membrane of the hair cell is equally accessible to TEA.

During stimulation, increasing receptor currents produce increasing depolarizations. However, given the voltage dependence of *g_KD_*, this enhances *g_KD_* activation and increases the potassium current driving force; both these events counteract the action of the receptor current. Thus, the amount of receptor current required to depolarize the cell by a fixed step increases with membrane depolarization; on the other hand, *g_KD_* also increases with membrane depolarization, and a fixed partial block cancels a similarly increasing amount of potassium current. Thus, independent of membrane potential—and the momentary level of activation of *g_REC_*—a fixed fractional block of *g_KD_* might produce a similarly sized depolarization, over a wide range of membrane potential.

In these experiments we measured the voltage-dependent potassium currents and estimated the size and reversal potential of a current through a lumped voltage-independent (“leak”) conductance. The computation of the zero-current potentials produced by variable amounts of transduction conductance, *g*_REC_, in the face of the measured currents, actually confirms the prediction that a fixed fractional block of voltage-dependent conductances produces a membrane potential shift whose magnitude is essentially constant, independent of the prevailing membrane potential, at least within the range −30 to −15 mV, a range which is supposed to comprise the actual peak depolarization during rotational stimulation of the intact labyrinth.

### Synaptic Transmission in Response to Hair Cell *V*_m_ Shift by TEA

We therefore employed a series of concentrations of TEA as an instrument to produce reasonably predictable shifts of membrane potential at the peak of rotational stimulation, and measured several parameters of quantal emission and junctional activity at the cytoneural junction of the posterior canal in the isolated frog labyrinth.

A series of concentrations of TEA, corresponding to predicted shifts in membrane potential ranging from a fraction of mV to about 7 mV, were employed at rest and during rotational stimulation. A reasonably linear relationship was detected between the magnitude of membrane potential shifts and the net increase in relevant parameters of transmitter release: peak quantal emission, cumulative quantal release during an excitatory episode, spike firing activity. The procedure proved a useful tool to quantitatively evaluate the sensitivity of key aspects of the synaptic machinery to membrane potential.

These experiments lead to the following general considerations: (1) Resting hair cells display an intense SA, presumably due to Ca^2+^ inflow into the partially depolarized cell, since a significant fraction of the transduction channel population is open when the cilia are in their resting position; (2) The overall mean rate of quantal release during stimulation is higher than at rest—although each excitatory hemicycle is followed by an equally intense inhibitory hemicycle—as if mechanical stimulation *per se* were able to activate release (see below); (3) Quantal emission at the cytoneural junction during stimulation (at least in the *V*_m_ range −30 to −15 mV) is linearly related to any small additive depolarization produced by the transduction current, over a relatively broad range of membrane potential; (4) A modest extra increase in mEPSP release is required to exceed action potential threshold and to activate the afferent fiber to transmit sensory information for central processing. This step is highly non-linear with respect to TEA-induced depolarization (or, presumably, the transduction current intensity) and is accompanied by large spatial-temporal summation in quantal emission; (5) For strong transduction current intensities the linear relationship between membrane potential and quantal release rate may be lost, because the depolarization may trespass the peak of the calcium I-V curve (Martini et al., 2000; Almanza et al., 2003; Bao et al., 2003); and (6) mEPSP peak amplitude is not affected in any consistent relevant manner by TEA, either at rest or during the excitatory/inhibitory hemicycles of rotatory stimulation.

The last-mentioned result appears to be in contrast with the data reported by Holt et al. (2006), who reported variable size and non-quantal behavior in the turtle posterior crista; however, in that preparation types I and II hair cells coexist and the complexity of intercellular contacts might favor the accumulation of potassium and glutamate and generate non-quantal activity. In the frog semicircular canals only type II hair cells with simple synaptic boutons are present and transmitter release displays a quantal behavior (Rossi et al., 1994). Entering further discussion of the mechanisms that might affect quantal size in the present experiments would be outside the target of this work.

### Estimating the Quantitative Relations Among Mechanical Stimulation, Hair Cell *V*_m_ Shift, Receptor Conductance and Transmitter Release

Some numerical examples might help to better define the general behavior of an ideal hair cell at the frog semicircular canal, as derived from the present, limited, observations. It should be stressed, however, that each parameter considered is affected by the large intrinsic variability among cells, and that modest quantitative modifications of one or more variables can easily generate the broad spectrum of physiological properties of single units observed *in vivo*.

Spontaneous synaptic activity, in the absence of stimulation, is present when the ciliary apparatus is in intermediate position at rest, and the resulting relatively high spontaneous afferent activity allows the hair cell to respond to stimuli in both directions, by either increasing or decreasing transmitter release. SA may also increase the sensitivity of the whole system, because the postjunctional membrane is displayed toward to the spike encoder threshold and even a small increase in stimulus intensity may markedly affect the frequency of spike discharge. These properties are readily verified at labyrinthine hair cells, where mEPSP emission can be examined.

Although the variability of quantal release is quite marked among units (about ±20% SE in the present control sample), the effects of mechanical stimulation and/or the application of drugs can be studied by focusing on the changes they produce in each cell; these changes are much more consistent and a tentative numerical description of the process can be attempted using the average values.

In our sample, the mean spontaneous quantal release at rest (cilia in resting position, partial depolarization) is about 210 ± 21 quanta/s; an average of 36 ± 8 quanta/s are added by stimulation (246/s ± 22/s average rate during the whole cycle), although the equivalent excitatory and inhibitory phases of stimulation should compensate each other. This might be due to some inactivation of K^+^ conductances during the depolarizing phase (Martini et al., 2009) or to the slow kinetics of Ca^2+^ disposal: the dynamics of intracellular calcium ion levels are complex (see, e.g., Scullin and Partridge, 2010); passive diffusion and buffering by Ca^2+^-binding proteins, uptake into the ER by the endoplasmic reticulum Ca-ATPase and extrusion across the cell membrane by the plasma membrane Ca^2+^-ATPase (neither Na:Ca nor Na:Ca, K exchanger operate in semicircular canal hair cells; Martini et al., 2002) contribute to restoring resting levels, while Ca^2+^-dependent Ca^2+^ release through ryanodine receptors and IP3-induced release from the endoplasmic reticulum might produce more persistent increases in Ca^2+^ levels, and transmitter release, at hair cells (Lelli et al., 2003; Rossi et al., 2006). In particular the average rate rises to about 282 ± 24 quanta/s (+73 ± 10 quanta/s vs. SA) during the excitatory hemicycle, and it attains 316 ± 24 quanta/s at the peak of the excitatory stimulation (+106 ± 10 quanta/s vs. spontaneous release). Conversely, during the inhibitory hemicycle the average rate declines to the same level as during the SA (210 ± 21 quanta/s) and it falls to 136/s at the peak of inhibition (−74/s vs. spontaneous release).

One may try to evaluate the effect of closing the receptor channels by subtracting the average release rate during the whole stimulation from the momentary rate at the peak of inhibition (136–246 = –110 quanta/s); if this quantity is subtracted from the SA, a rate of some 100 quanta/s remains. This might suggest that the stimulus intensity here used was not sufficient to close all the channels; alternatively, the closure of receptor channels during the inhibitory cycle, even if it were complete, was only transient, and therefore possibly less effective (see also the above considerations about possible “residual” Ca^2+^).

### Quantitative Considerations on Sensory Input Convergence

The extra release of about 360 quanta during the 5-s excitatory hemicycle (compared to the 1045 quanta that would have been released spontaneously during the same period) appears to be sufficient to generate the afferent information for central processing.

The gross organization of the posterior semicircular canal in the frog is constituted by about 4,000 hair cells in the sensory epithelium of the whole crista (data derived from Russo et al., 2007), innervated by some 600 afferent fibers (Taglietti et al., 1973). To a first approximation, a single fiber should contact a mean of 6–7 hair cells. The ampullar nerve contains a fraction (≈10%) of large myelinated fibers, larger than 8 μm, which most likely are the ones impaled by the microelectrode. As described in the bullfrog (Honrubia et al., 1989), in frog the thick afferent fibers turn toward the more central region of the crista and receive little convergence of afferent impulses (Taglietti et al., 1973). Thus, the sensory units considered here are comprised of few hair cells, arranged in parallel, with relatively homogeneous biophysical properties, as known from patch-clamp analysis. The functional variability observed in individual primary fibers is likely due to the innervation pattern and to the properties of the encoder system of each fiber, rather than to the transduction mechanism itself, as originally suggested by Precht et al. (1971). From our computations, each nS of transduction current should produce a shift in membrane potential of about 1.6 mV (at −30 mV membrane potential) or 0.29 mV (at −15 mV), and consequently contribute about 7–41 quanta/s (at −15 or −30 mV, respectively) to an afferent fiber, i.e., an additional release of 1–6 quanta/s per hair cell. This would correspond to the emission of about 55–300 quanta (8–45 per hair cell) during the 5-s excitatory hemicycle analyzed here.

### This Approach can only be Applied to Depolarized Hair Cells

One may wonder why we did not simply examine the effects of TEA on quantal release in the resting preparation, rather than during mechanical stimulation. The resting hair cell presumably is sitting in a region below the threshold of TEA sensitive currents (Figures 3D, 4A) and where the shift of *V*_m_ produced by a given concentration of TEA is far from constant (Figure 4C). There, the effect of TEA on membrane potential is less predictable (see, in Figure 4, how the shift in membrane potential produced by TEA becomes highly voltage-dependent for membrane potentials more negative than –30 mV), and its effects on quantal release cannot be related to a reliably estimated membrane potential shift (and to a corresponding change in receptor conductance). Actually, quantal release in the resting preparations was affected in a variable way by TEA (data for 1 mM TEA in five units are reported in Table 2, but a dose effect curve was not even attempted in a larger sample).

In the region of potassium current activation, the null-point of the interaction between the receptor current and potassium currents (mostly IKD) determines the zero-current potential of the hair cell membrane and consequently the quantal emission at the cytoneural junction. Independent of the variability among single cells, a relevant control mechanism in each cell consists in the time- and voltage-dependent process of *g_KD_* inactivation, which can modify maximum amplitude of IKD by a factor up to 1.8, if the cell is held at −100 mV instead of the typical −40 mV potential in the resting preparation, or 1.2 if it is held at −70 mV. Independent of the steady-state inactivation mechanism, IKD is a quite strong current (several nA) and requires a similarly strong transduction current to be generated in order to have significant effects at the cytoneural junction. The range of possible transduction conductances considered in the present paper (up to 20 nS) exceeds the values measured in various hair cells or assumed in cell models (0.65–7.5 nS: Holton and Hudspeth, 1986; Soto et al., 2002; Zeddies and Siegel, 2004; Neiman et al., 2011).

Provided the I-V curve for IKD is not modified in shape but only scaled down by TEA, increased transduction conductance will shift the null-point towards more positive potentials (Figure 4). Hair cell synapses appear to operate with a linear Ca^2^-dependence of release, as shown in the frog papilla (Keen and Hudspeth, 2006), in mouse cochlea (Brandt et al., 2005; Johnson et al., 2005) and turtle papilla (Schnee et al., 2005) by using capacitance recordings. Since above a certain *V*_m_ calcium current stops increasing and rather decreases (the I-V for ICa displays a maximum between −25 and −10 mV), our observations on quantal release suggest we are not pushing the membrane potential above the peak of Calcium current I-V peak. Receptor potentials, occasionally larger than 15 mV, were evoked in bull frog saccular hair cells by direct mechanical stimulation of their hair bundle in the excitatory direction. Similarly large transient depolarizations were recorded in vestibular hair cells of the chick, following mechanical stereociliary shift: their amplitude was 24 mV at −43 mV holding potential and 30 mV at −70 mV holding potential (Hudspeth and Corey, 1977; Ohmori, 1985). Under the rotatory stimulation used in the present experiments, the maximum calcium inflow occurs at about −25 mV (see Figure 8 in Rossi et al., 2010); since hair cells likely exhibit a resting potential of about −45 mV, the voltage range over which calcium entry in expected to rise linearly and feed a linear increase in the quantal emission rate, is restricted to a 20 mV range, included within the rising branch of the ICa I-V curve. Negative to −45 mV, the calcium channels are progressively closed (the ICa activation curve starts at −50/−52 mV), and above −25 mV calcium inflow significantly decreases (Rossi et al., 2010), thereby shunting the effects of increased transduction current on transmitter release: possible further depolarization is not expected to produce additional quantal emission.

By applying TEA at high concentrations we should have changed the membrane potential, at the peak of excitation during a rotational stimulus, by up to 7 mV; this would correspond to an extra transduction conductance slightly above 3 nS (if the membrane potential were −30 mV) or up to 17 nS (for −15 mV membrane potential). We do not know whether such large receptor conductances can be generated in frog hair cells. The maximum transducer conductance estimated by Crawford et al. (1991) and Kros (1996) was around 13 nS; from our data and computations, a linear relation should hold between quantal emission and transducer conductance up to this value. Above this value, however, and above −10 mV in particular, calcium inflow, and quantal emission, may even decrease.

### Dynamic Changes During Rotational Stimulation

A final consideration must be dedicated to a possible problem raised by mechanical stimulation. Quantal release as well as spike firing at the cytoneural junction displays marked hysteresis, i.e., an asymmetry between the rising and falling phases of mechanical stimulation (e.g., Rossi et al., 1989, 2010); similarly, the current profiles at the hair cell are sensitive to the previous history of membrane potential, because complex processes of time- and voltage-dependent inactivation, and recovery from inactivation, influence the availability of channels sustaining IA as well as IKD (Martini et al., 2009); this produces asymmetric current time-courses when the hair cell is driven along a sinusoidal voltage path. These observations have been confirmed in this study, and similar asymmetry is shown to affect the TEA-sensitive fraction of K currents, which is in agreement with previous observations about inactivation of delayed K^+^ currents (Martini et al., 2009); such asymmetry, however, is quantitatively limited (4–19%) and does not interfere with the main aspect here studied, namely the properties of the hair cell and junctional transmission at the peak of excitatory stimulation (or during the whole excitatory hemicycle, so that possible asymmetries in rising and falling phases cancel each other out).

## Conflict of Interest Statement

The authors declare that the research was conducted in the absence of any commercial or financial relationships that could be construed as a potential conflict of interest.
